# Characterization of labiomandibular movements induced after isolated LeFort I osteotomy in the surgical management of class III malocclusion

**DOI:** 10.1371/journal.pone.0292391

**Published:** 2023-10-09

**Authors:** Chris Passalboni, Maxime Taverne, Raphael Lopez, Maxime Rotenberg, Frédéric Lauwers, Alice Prevost

**Affiliations:** 1 Plastic and Maxillo-facial Surgery Department, Toulouse University Hospital Center, Toulouse, France; 2 Craniofacial Growth and Form Laboratory, Necker-Enfants Malades Hospital, Assistance Publique-Hôpitaux de Paris, Paris, France; 3 Dental Faculty, Department of Orthodontics, Toulouse University Hospital Center, Toulouse, France; University of the Pacific Arthur A Dugoni School of Dentistry, UNITED STATES

## Abstract

**Introduction:**

Maxillary surgery alone can be proposed for the surgical management of class III malocclusion, but anticipating outcomes for the labiomental muscle complex is challenging due to the mandibular autorotation phenomenon. The objective of this study was to quantify the mandibular and labiomental movements induced by maxillary osteotomy alone in the management of class III malocclusion according to different clinical and surgical variables.

**Methods:**

The post-operative changes in mandibular and labiomental shapes were studied by geometric morphometry from the pre- and post-operative lateral cephalometric radiograph of 25 patients. The explanatory variables tested were maxillary advancement, maxillary rotation, and divergence.

**Results:**

Soft tissues repositioning are different from postoperative mandibular repositioning after maxillary osteotomy. Neuromuscular adjustments of mandible depend on divergence and the maxillary rotation. Labiomental response only depends on divergence.

**Conclusion:**

The surgical procedure does not have the same bone-related and musculocutaneous effects on patients with the same class III malocclusion. It is therefore essential for surgeons to understand the effects of their procedure on musculocutaneous tissues in order to best anticipate post-operative outcomes.

## Introduction

Class III malocclusion can manifest in different clinical forms and be associated with varying degrees of sagittal and vertical, maxillary and mandibular abnormalities [1]. Morphometric studies associated with principal component analysis have quantified and defined many phenotypic sub-groups reflecting the large variations in this malocclusion [2–4] that could help in guiding us towards the most appropriate choice of surgical technique [2–4]. In fact, the surgical management of class III malocclusion varies depending on the nature of the bone abnormalities and authors, but to date no official guidelines have been established [5]. Surgeons can perform mandibular setback surgery, maxillary advancement surgery, or combined surgery (maxillary advancement and mandibular setback). It is now recognized that occlusal stability after mandibular setback surgery alone is inferior to combined surgery [6–10] and maxillary advancement surgery alone is a stable procedure for correction [11]. Finally, maxillo-mandibular surgery does not appear to be clinically superior to maxillary advancement surgery alone [12–14]. In contrast, a recent study has demonstrated that post-operative morbidity, intervention duration, and hospitalization length were higher after maxillo-mandibular surgery than maxillary surgery alone [15].

In addition to occlusal stability and operative morbidity, the surgeon must also take into account patient satisfaction with post-operative morphology. Indeed, the reason patients agree to orthodontic surgical management lie in the resulting morphological changes, and not in the occlusal or functional changes [16, 17]. Anticipation of post-operative changes to soft tissues is therefore essential, but reliably predicting them still remains a challenge [18–20].

The mandibular autorotation phenomenon (MAP), which must be taken into account during any maxillary surgery, is still poorly understood as demonstrated by the limited number of reports found in the literature. However, Chang et al. [20] indicate that differences between study findings could be explained in part by the fact that the centre of mandibular rotation is still only a theoretical idea; the precise definition and location still thus remaining author dependant. Nonetheless, several studies suggest that any maxillary impaction induces chin impaction and advancement [21–23]. Maxillary impaction therefore tends to reduce facial height at the expense of increasing sagittal length, thus resulting in aggravation in progenia and often considered unattractive [24–26]. The profile tends to be more convex, or "transfrontal" in patients who complain mainly of "over-prominent" chins. In this light, we must reflect upon the relevance of proposing maxillary surgery alone to patients with class III malocclusion. Any risks of aggravating progenia should be considered and carefully quantified.

The objective of our study was therefore to quantify the labial and mandibular movements induced by maxillary osteotomy alone in the management of class III malocclusion according to different clinical and surgical variables. We tested how post-operative mandibular position correlated with maxillary advancement (H1), maxillary impaction movement (H2), and/or individual divergence profiles (H3). We also investigated how post-operative changes in labiomental morphology correlated with maxillary advancement (H4), maxillary impaction movement (H5), and/or individual divergence profiles (H6).

## Materials and methods

### 1. Study design and patients

We carried out a retrospective, single-center, cohort study from 1^st^ January 2013 until 31th March 2022 (10 years and 3 months). We included all patients that had undergone an isolated LeFort I osteotomy in our Plastic and Maxillo-facial Surgery Department (Toulouse University Hospital) to correct a class III malocclusion as defined by ANB angle (below 0°) [27]. Patients were only included if their radiographic assessment included a pre- and post-operative lateral cephalometric radiograph (LCR) carried out less than six weeks after the intervention. The LCR was carried out using occlusion with the lips at rest. We excluded patients with: indication of LeFort I osteotomy due to trauma, maxillo-mandibular malocclusion related to a malformation syndrome, and individuals with obstructive sleep apnea syndrome.

An official waiver of ethical approval was granted from the IRB of Toulouse University Hospital given the retrospective nature of the study as asserted by French Jardé law (study reference RnIPH 2023–07). All the procedures performed were part of routine care, and both in accordance with institutional guidelines and with the principles and regulations of the Declaration of Helsinki. Informed patient consent was obtained from all participants and all data has been anonymized for publication purposes.

### 2. Surgical technique

The intervention consisted of a LeFort I osteotomy [28], a standardized surgical technique in our center and therefore performed in the same way by all surgeons. The osteotomy was carried out with a piezoelectric scalpel from the piriform orifices up to the pterygomaxillary arches. In the event of impaction, a second upper osteotomy line was executed; the height depended on the pre-operative plan. Osteosynthesis was ensured by the insertion of two plates on each side: one on the canine pillar and the other on the maxillo-zygomatic arch after the execution of a maxillo-mandibular block with steel wire. The final block was carried out with elastics using inter-maxillary traction III (TIM III). The procedure was carried out in outpatient hospitalization or during a short hospital stay.

### 3. Definition of landmarks

Given the primary study objective was to determine the labial and mandibular movements induced by maxillary surgery, we studied post-operative changes in mandible position and lower lip shape determined by geometric morphometry from LCR. We considered post-operative lower labial edema to be clinically insignificant after maxillary surgery alone.

Geometric morphometry (GM) is based on the acquisition, processing, and analysis of landmarks placed at key anatomical locations on objects to quantify morphological variation, while excluding biases related to position, orientation, and scale [29].

Landmarks can be categorized into "anatomical" landmarks and semilandmarks [30]. Anatomical landmarks (LM) are discrete anatomical loci that are biologically homologous. A semilandmark (SLM) refers to a geometrically constructed point that is not necessarily supported by homology. Furthermore, Weber and Bookstein [31] defined three types of SL: SL on curves, SL on surfaces, and constructed SL (i.e. at the beginning and end of a curve).

**Fig 1:**
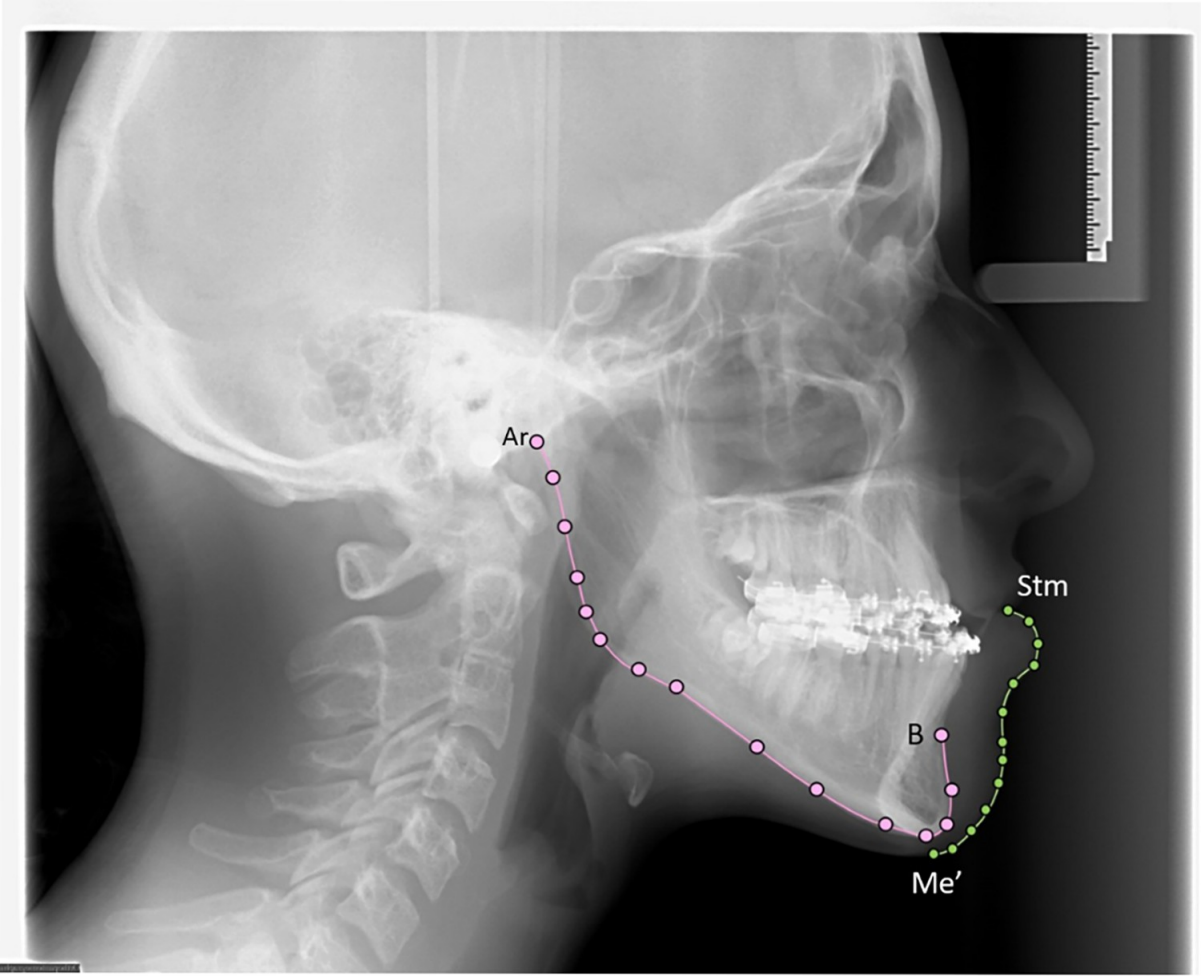
Mandibular (pink) and labio-mental (green) curves.

We defined a set of LMs (based on cephalometric analysis) and SLs on curves that were placed on the lateral cephalometric radiographs of each patient from our sample (Fig 1). To ensure geometric comparability between the corresponding SLs, these were slid using a thin-plate spline (TPS) algorithm that minimized bending energy [32, 33]. The placement of the LMs, as well as the semi-automatic measurements of the SL on the lower and mandibular labial curves using the TPS algorithm, were carried out using the Viewbox Cephalometric Software (version 4.1, Dhal, Athens, Greece).

We used four anatomical LMs (Fig 1): the Ar point (posterior articular, intersection of the posterior edge of the Ramus with the exocranial surface of the clivus), B (Supramental, the most sloping point of the anterior mandibular alveolar concavity), the Stomion point (Stm) (junction between the upper and lower lips), and the soft tissue chin point (Me’) (soft tissue projection of the bony chin point, perpendicular to the Frankfort plane).

The mandibular curve was defined by 15 SLM, between the points Ar and B. The labio-mental shape was characterized by a second curve, defined by 13 SLM placed between Sto and Me’ (Fig 1).

The mandibular and labio-mental shapes were therefore characterized by the Cartesian coordinates of these SLM.

The repeatability (i.e. intra-operator error test) of the measurements was assessed using the intraclass correlation coefficient (ICC, two-way mixed-effects model, perfect agreement) [34]. Five lateral cephalometric radiographs were used to assess the repeatability of the measurements. One operator (AP) positioned the anatomical landmarks (Arp-B-Sto-Me’) on these five radiographs three times/week at one-week intervals. ICC was 0.931–0.99 for the different measurements studied, indicating an excellent repeatability.

### 4. Explanatory variables

Data on three explanatory variables were collected for each patient and tested as potential predictors for post-operative changes in mandibular and labio-mental morphology: maxillary advancement, maxillary impaction, and divergence.

Maxillary advancement (in mm) was characterized by the difference between the pre- and post-operative coordinates (x;y) of the ANS (anterior nasal spine) point on the lateral radiographs. For maxillary impaction, we distinguished "clockwise maxillary impaction" (when posterior impaction was more significant), "anti-clockwise maxillary impaction" (when anterior impaction was more significant), and "homogenous maxillary impaction" (when both impactions were similar). The anterior and posterior impactions were characterized by the difference between the pre- and post-operative coordinates (x;y) of the ANS and PNS (posterior nasal spine) points on the lateral radiographs. Finally, divergence was defined by the Frankfurt-mandibular plane angle (FMA, angle formed by the intersection of the Frankfort horizontal plane with the mandibular plane). A patient was considered hypo-divergent if FMA was <22°, normo-divergent if FMA was 22–28°, and hyper-divergent if FMA was >28°.

Since diagnostic cephalometry enables the study of skeletal malocclusion, divergence and characterization of maxillary movements were carried out using the Viewbox Cephalometric Software.

### 5. Statistical analyzes

The SLM coordinates defining the lower lip and mandibular shapes were then aligned by Procrustes superimposition. The resulting Procrustes (x,y,z) coordinates were extracted and used as input variables in all subsequent analyzes [29, 35]. P-values below 0.05 were considered statistically significant.

The effect of maxillary surgery on mandibular morphology changes was confirmed by an Anova with repeated measurements (n = 10000) for the Mahalanobis distances between the "pre-operative mandible" and "post-operative mandible" groups. We repeated this for the "pre-operative lip" and "post-operative lip" groups.

A Two-Block Partial Least Squares regression (2b-PLS) was computed to test for covariation between pre-operative (first block) and post-operative (second block) mandibular shape. The influence of divergence and maxillary movement (H2 and H3) were assessed by examining how groups were discriminated along the PLS axis. Another 2b-PLS regression was computed to test for covariation between pre-operative (first block) and post-operative (second block) labiomental shape. The influence of divergence and maxillary movement (H5 and H6) were assessed by examining how groups were discriminated along the PLS axis. When PLS axes revealed some differences between groups, a canonical analysis of the variables was ran on the co-variance matrix of shape (pre- and post-operative) coordinates in sub-groups for the different variables [36]. The effect of maxillary advancement was investigated by regression analysis against the canonical variable in pre- and post-operative groups (to test H1 and H4).

Procrustes superimposition and statistical analyzes were carried out using the MorphoJ software [37] (version 1.07a, Manchester–United Kingdom). Theoretical shapes resulting from all analyzes are presented using WireGraph configurations.

## Results

### 1. Cohort characteristics

Twenty-five patients were included: 12 females and 13 males with an average age of 24.6 years (±10.58 years). Surgeries were performed by three different surgeons but the majority (76%) of the procedures were performed by the same surgeon. The post-operative lateral cephalometric radiographs were carried out on average 8.5 months (±7 months) after the surgery. Data for the different variables studied are detailed in Table 1.

**Table 1:** Description of the variables studied for the whole cohort (n = 25) after surgery.

Maxillary advancement in *mm* *(±SD)*	4.8 (± 1.9)
**Maxillary impaction *n (%)***	
Clockwise rotation	11 (42.3)
**Anti-clockwise rotation**	8 (36.6)
**Homogenous impaction**	6 (23.1)
**Divergence *n (%)***	
Hypo-divergent	7 (26.9)
Normo-divergent	12 (46.2)
Hyper-divergent	6 (26.9)

SD: standard deviation; mm: millimeter; n: number; %: percentage

### 2. Quantification of changes in mandibular morphology after LeFort I osteotomy

Multivariate analyzes of variance indicated that mandibular shape was significantly different after surgery (p <0.0001).

#### a. Relationship between divergence and post-operative mandibular shape

**Fig 2:**
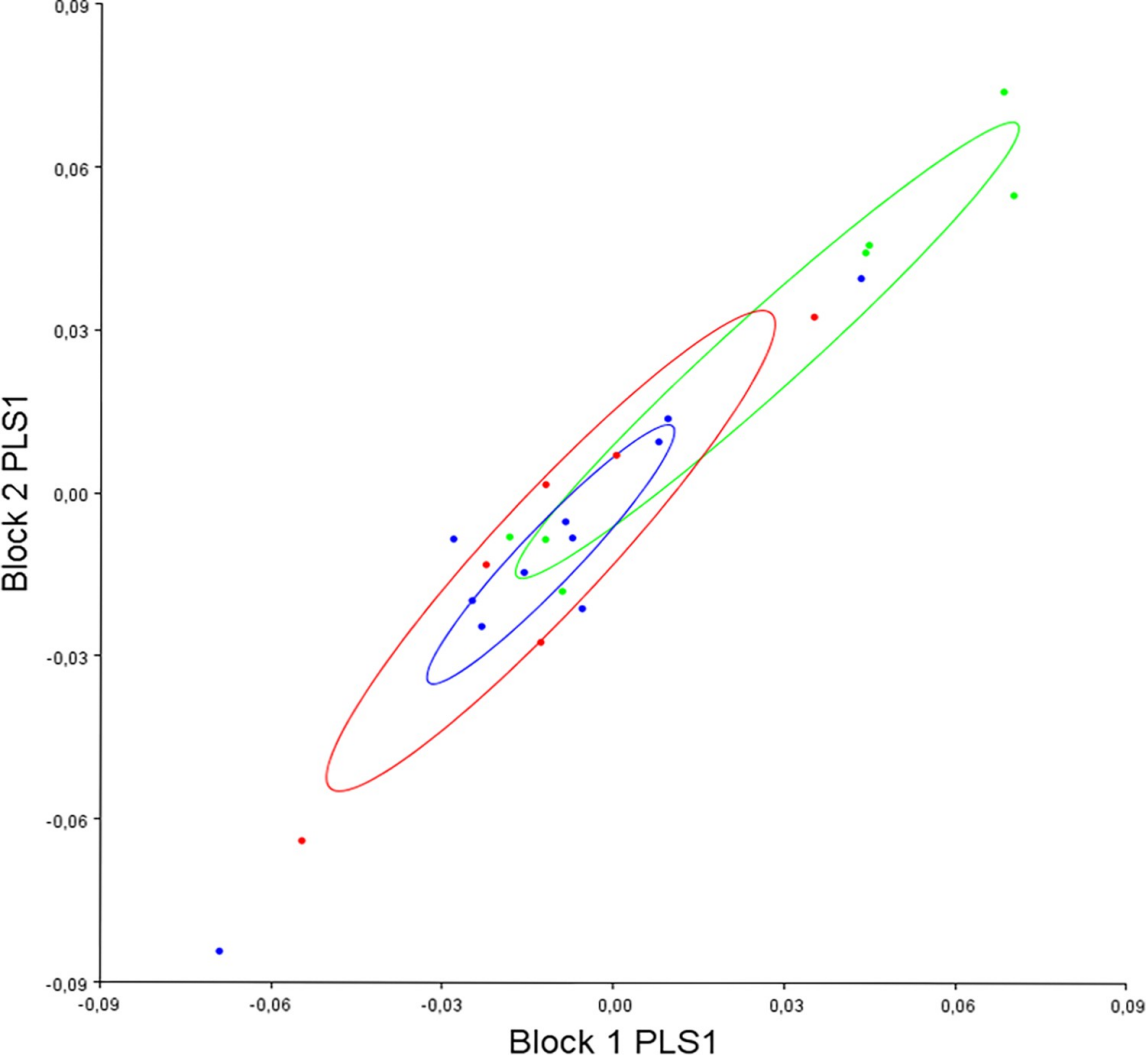
Distribution of all individuals according to PLS 1 divergence (block 1: Pre-operative mandibular shape, block 2: Post-operative mandibular shape). Red: hyper-divergent; green: hypo-divergent; blue: normo-divergent.

The 2b-PLS regression revealed that pre- (block 1) and post-operative (block 2) mandibular shape strongly covaried (r: 0.86, p<0.001) and appeared to evolve according to PLS 1 divergence (which represented 67.87% of the co-variation) (Fig 2).

**Fig 3:**
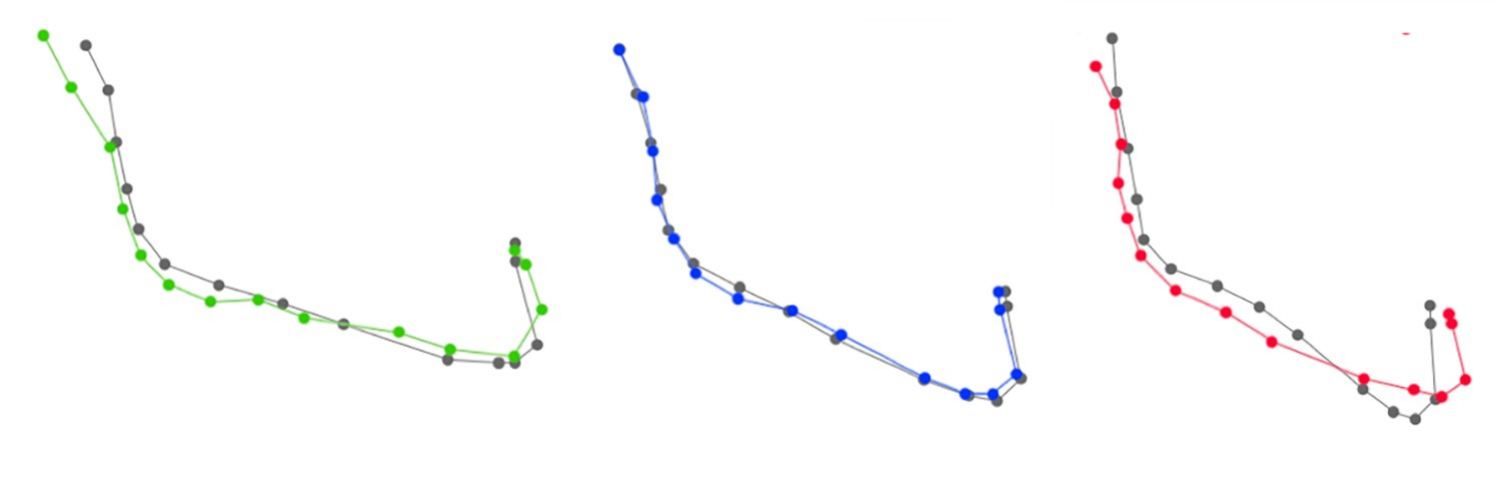
Mandibular autorotation after maxillary surgery depending on type of divergence. Green: hypo-divergent; blue: normo-divergent, red: hyper-divergent (CV score 10). Gray is the corresponding preoperative form.

We therefore analyzed these divergence-related changes in mandibular shape by carrying out a canonical analysis of pre-operative and post-operative variables in the different sub-groups of divergence (Fig 3).

In hypo-divergent patients, we noted a slight symphyseal advancement with no associated vertical movement (CVA pre-op/post-op; p = 0.038). The changes were more pronounced for hyper-divergent patients. We noted a reduction in gonial angle and symphyseal ascension and advancement (CVA pre-op/post-op; p<0.001). There were no significant changes among normo-divergent patients (CVA pre-op/post-op; p = 0.74).

### 3. The effect of maxillary advancement on changes in mandibular shape

Maxillary advancement had no effect on mandibular shape (p = 0.4, % predicted = 1.3%) regardless of the patient divergence profile (Table 2) (multivariate regression analysis: dependent variable CV1, independent variable advancement).

**Table 2:** Effect of maxillary advancement on changes mandibular shape according to divergence.

Divergence	% predicted	p
Hyper-divergent	9.1	0.33
Hypo-divergent	0.2	0.9
Normo-divergent	0.09	0.9

### 4. The effect of maxillary impaction on changes in mandibular shape

**Fig 4:**
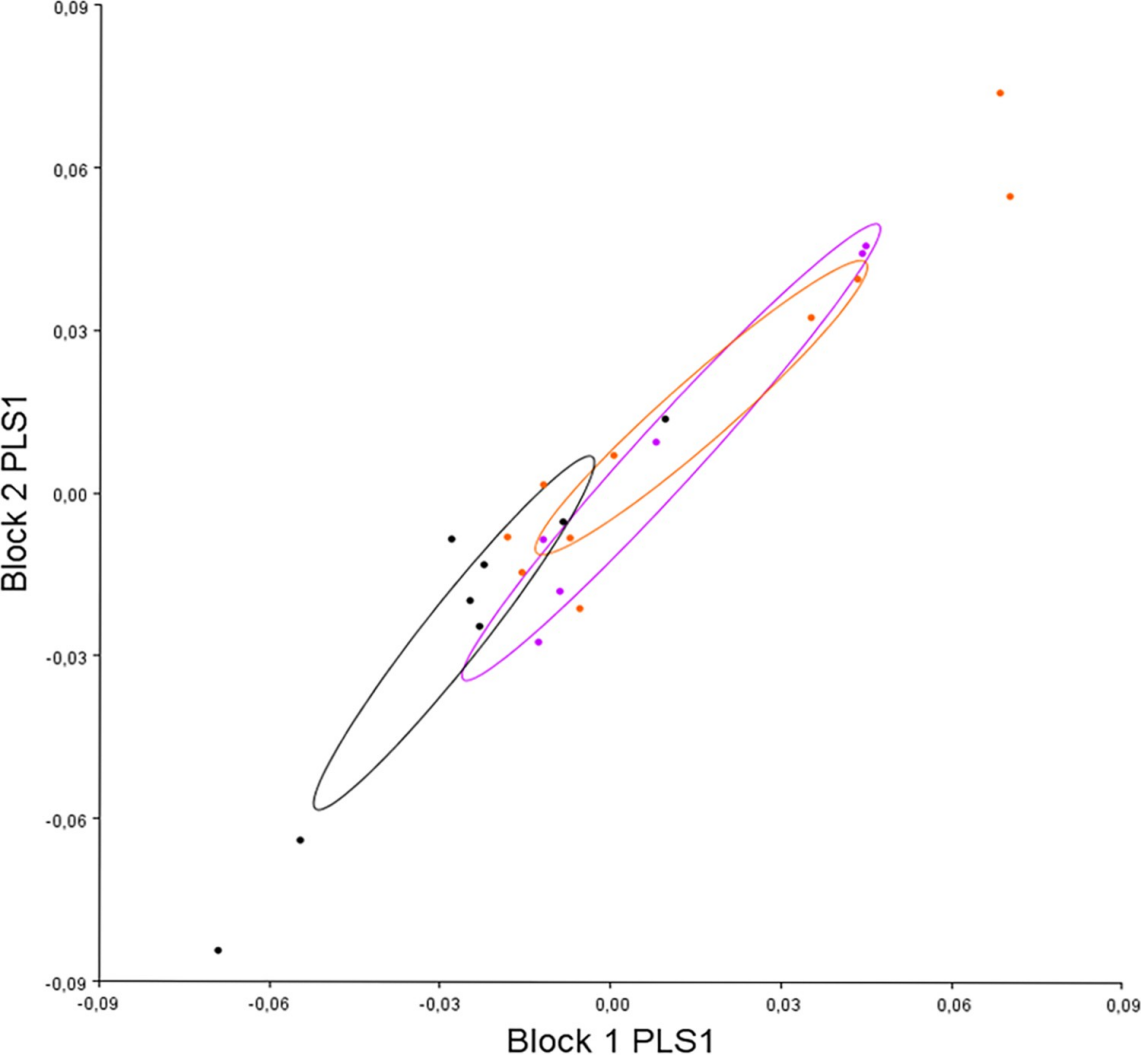
Distribution of all individuals on PLS 1 according to surgical maxillary impaction (block 1: Pre-operative mandibular shape, block 2: Post-operative mandibular shape). Black: anti-clockwise; purple: homogenous; pink: clockwise).

Co-variance analysis between pre- and post-operative mandibular shapes suggests that both shapes evolve according to surgical maxillary movement (Fig 4).

**Fig 5:**
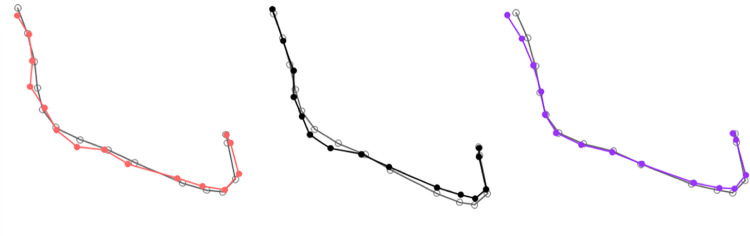
Mandibular rotation according to maxillary impaction. Black: anti-clockwise; purple: homogenous; pink: clockwise (CV score 10). Gray is the corresponding preoperative form.

We therefore analyzed changes in mandibular shape according to maxillary impaction movement by carrying out a canonical analysis of pre-operative and post-operative variables. We found changes in mandibular position when clockwise maxillary impaction was performed (p<0.0001). Clockwise maxillary impaction appeared to induce symphyseal advancement and ascension while the height of the mandibular angle remains similar. There were also active changes in mandibular position when anti-clockwise maxillary impaction was performed (p<0.0004). Anti-clockwise maxillary impaction induced symphyseal ascension without advancement and a reduction in mandibular angle. Homogenous maxillary impaction did not induce significant mandibular repositioning (p = 0.9) (Fig 5).

The mandibular autorotation phenomenon varied according to divergence and maxillary impaction movement.

### 5. Quantification of morphological changes in the lower lip after LeFort I osteotomy

There was a difference in morphology between the pre-operative and post-operative lower lip shapes. Maxillary surgery therefore affects lower lip shape (ANOVA, p <0.0001).

#### a. The effect of divergence on changes in post-operative lower lip shape

**Fig 6:**
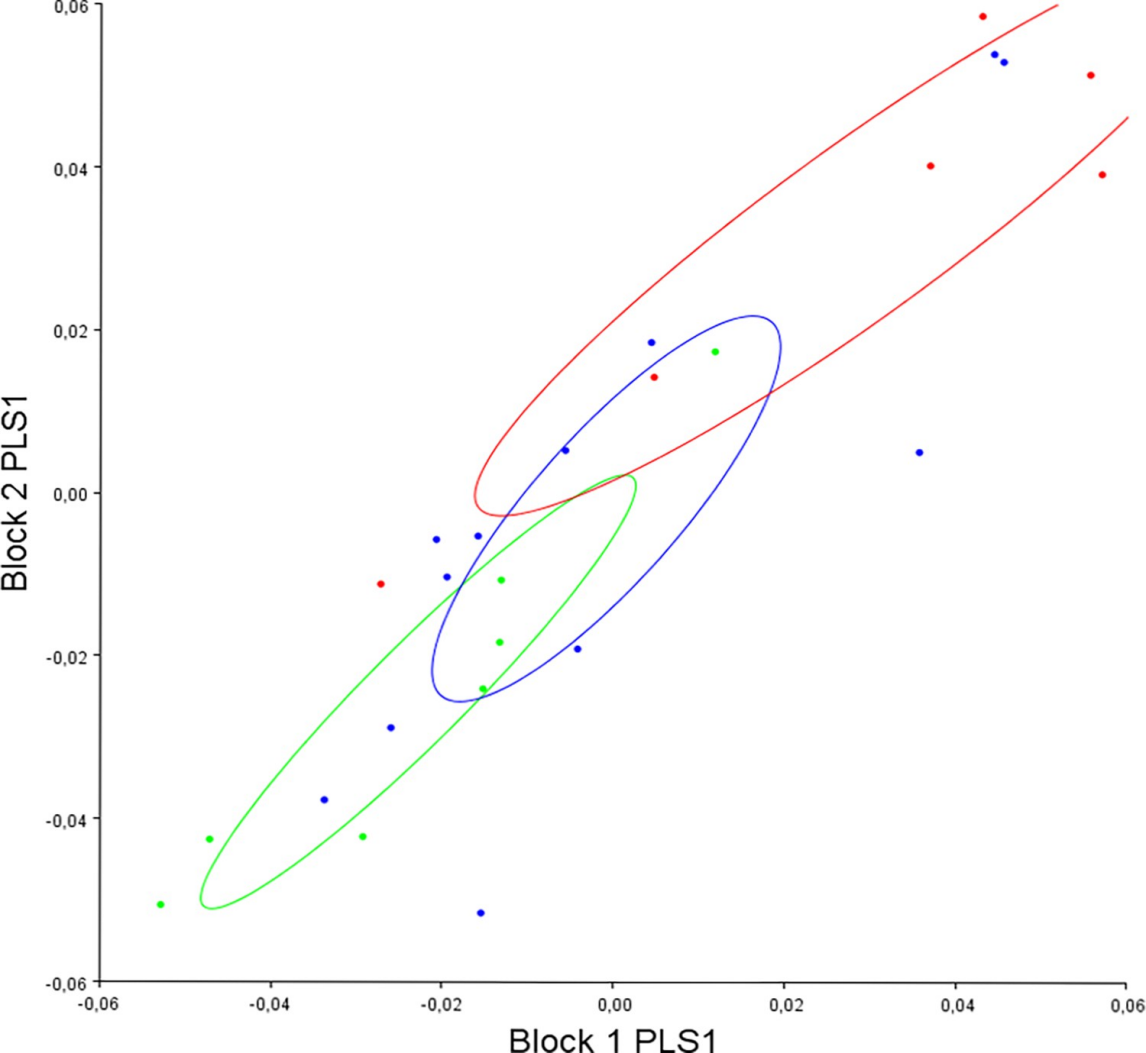
Distribution of all individuals according to divergence on PLS 1 (block 1: Pre-operative labio-mental shape, block 2: Post-operative labio-mental shape). Red: hyper-divergent; green: hypo-divergent; blue: normo-divergent.

The PLS analysis enabling the study of variation in pre- (block 1) and post-operative (block 2) labio-mental shapes confirmed that these shapes were correlated (r: 0.76, p<0.001) and that they appeared to evolve according to PLS 1 divergence (which represented 59.8% of the co-variation) (Fig 6).

**Fig 7:**
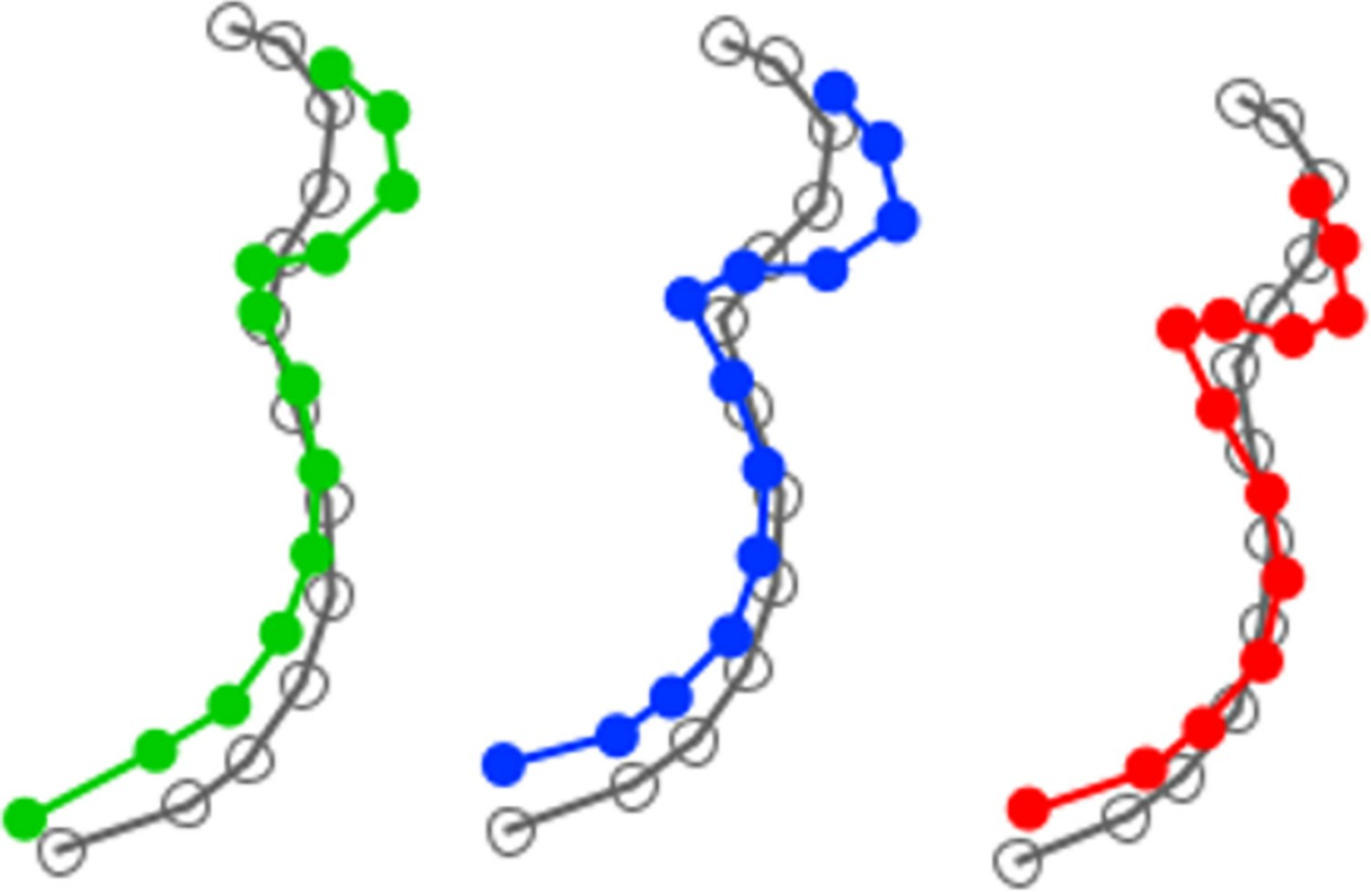
Changes in lower lip shape according to divergence. Green: hypo-divergent; blue: normo-divergent, red: hyper-divergent (CV score 20). Gray is the corresponding preoperative form.

We therefore analyzed changes in post-operative lower lip shape according to divergence by carrying out a canonical analysis of pre-operative and post-operative variables in the different sub-groups of divergence (Fig 7).

In hypo-divergent patients, maxillary surgery appeared to induce a push back of the soft tissue of the chin and red lip protrusion (p<0.0001). Maxillary surgery induced similar but more marked changes in hyper-divergent patients. We noted a more pronounced labio-mental fold (p = 0.0015) a reduction in labio-mental height. The soft tissue of the chin was pushed back with red lip protrusion in normo-divergent patients (p<0.0001).

#### b. The effect of maxillary advancement on changes in lower lip shape

Maxillary advancement had no effect on changes in labio-mental shape (p = 0.24, % predicted = 2.89%) regardless of patient divergence profile (Table 3) (multivariate regression analysis: dependent variable CV1, independent variable advancement).

**Table 3:** Effect of maxillary advancement on changes in labio-mental shape according to divergence.

Divergence	% predicted	p
Hyper-divergent	0.03	0.95
Hypo-divergent	0.16	0.89
Normo-divergent	0.25	0.81

#### c. The effect of maxillary impaction on changes in lower lip shape

**Fig 8:**
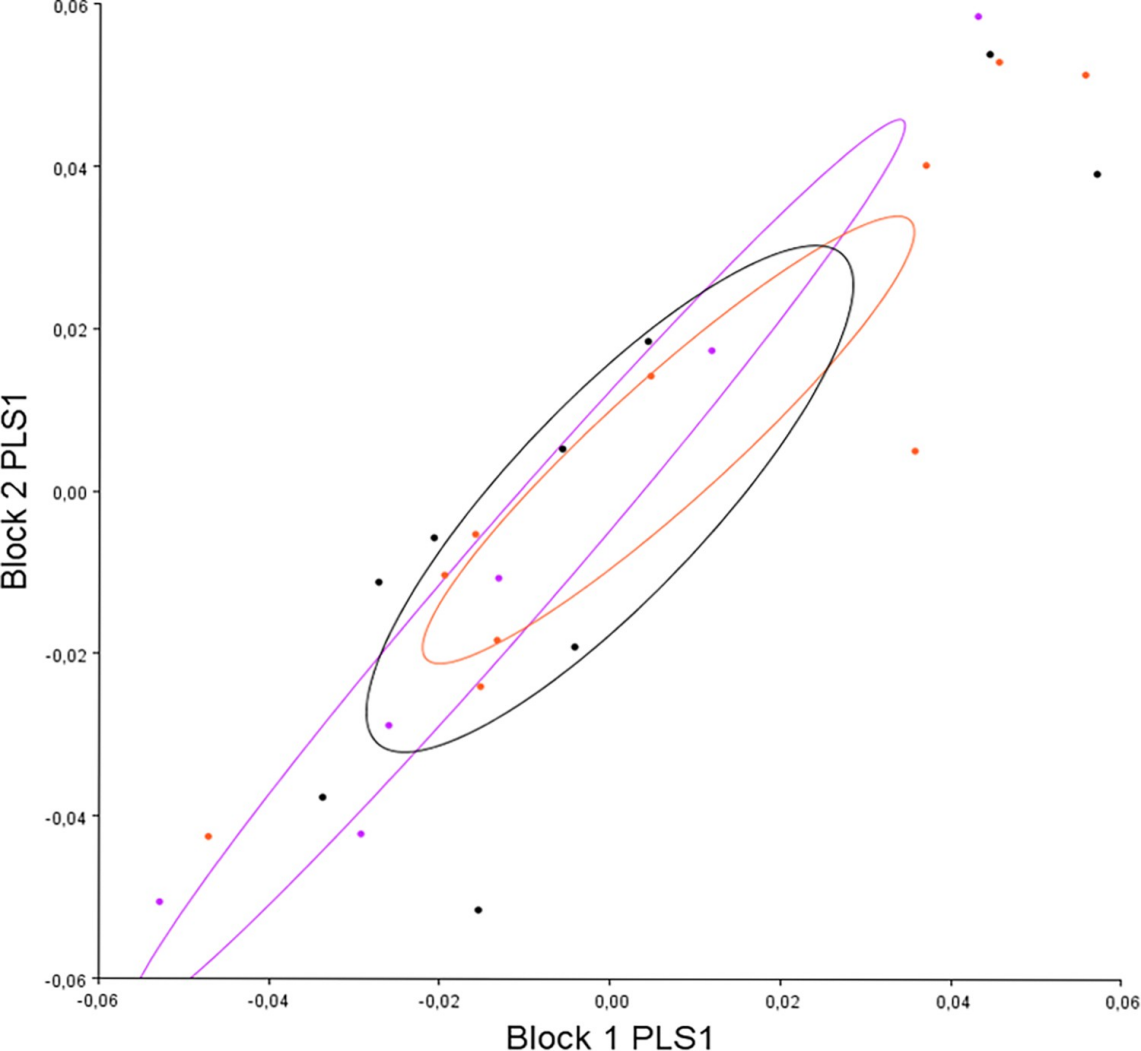
Distribution of all individuals according to surgical maxillary movement on PLS 1 (block 1: Pre-operative labio-mental shape, block 2: Post-operative labio-mental shape). Black: anti-clockwise; purple: homogenous; pink: clockwise).

The PLS analysis enabling the study of the variation in pre- (block 1) and post-operative (block 2) labio-mental shapes showed that surgical movement did not underlie the correlation between pre- and post-operative labio-mental shapes (Fig 8).

In all, only divergence significantly affected post-operative lower lip shape.

### 6. Correlation between pre-operative mandibular and post-operative labio-mental shapes

**Fig 9:**
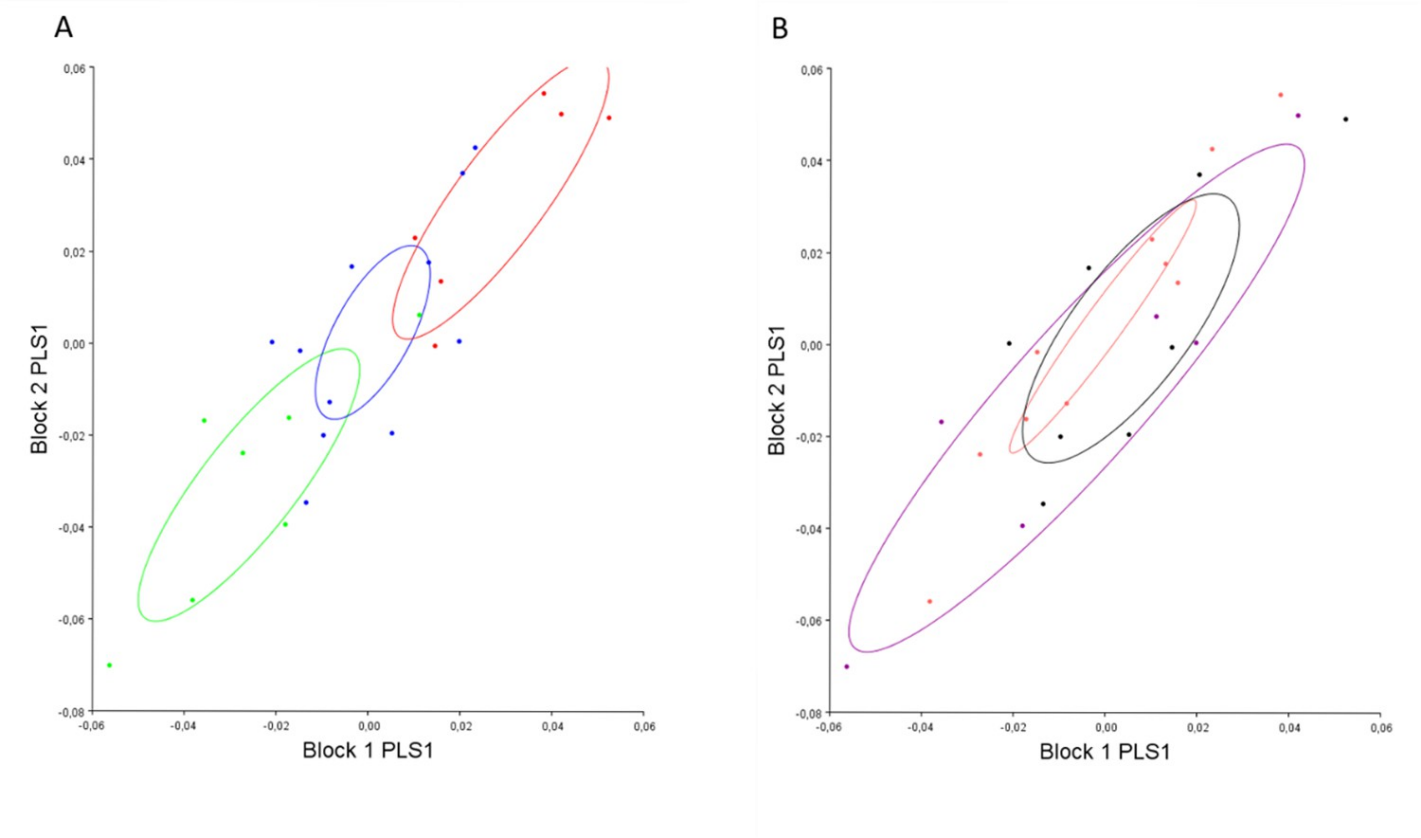
Co-variation between pre-operative mandibular shape (block 1) and post-operative lip shape (block 2) according to divergence (A) and surgical maxillary movement (B). A: Red: hyper-divergent; green: hypo-divergent; blue: normo-divergent. B: black: anti-clockwise; purple: homogenous; pink: clockwise.

We carried out co-variation analysis (PLS) between pre-operative mandibular shape (block 1) and post-operative labio-mental shape (block 2); we found co-variation between them (coef 0.36 p = 0.002). Analysis of the distribution of individuals on PLS 1 (which represented 68.52% of the co-variation) showed that divergence underlies this correlation (Fig 9A) contrary to surgical movement (Fig 9B).

**Fig 10:**
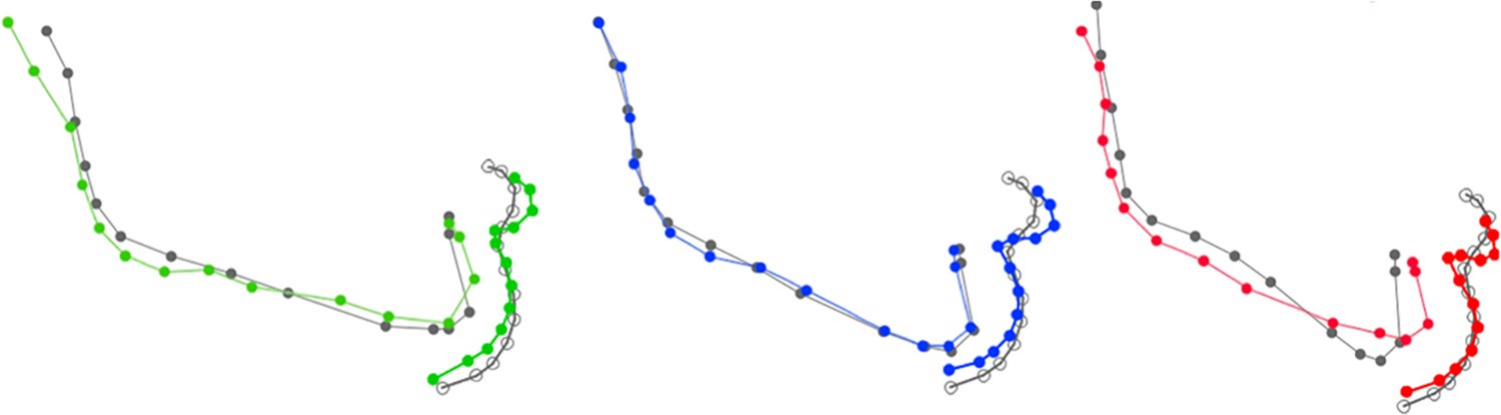
Summary of post-operative shape changes from pre-operative shape (gray) according to divergence. Green: hypo-divergent; blue: normo-divergent, red: hyper-divergent.

Among the explanatory variables we studied, only divergence seemed to cause the changes in shape (Fig 10). Mandibular advancement did not induce soft tissue advancement of the chin in hypo-divergent patients. Only the red lip protruded. The same observations were found in hyper-divergent patients. Despite significant mandibular advancement, the soft tissue of the chin did not move. In normo-divergent patients, maxillary surgery did not induce any clear changes in mandibular position, but the adjacent soft tissues underwent a slight mandibular retrusion. We noted that the more advanced post-operative mandibular point B, the more accentuated the labio-mental folds.

## Discussion

The objective of our study was to quantify the mandibular and labio-mental movements induced by maxillary osteotomy alone in the management of class III skeletal malocclusion. By testing a series of explanatory factors, we demonstrate here that changes in post-operative mandibular position were not dependent on the extent of the maxillary advancement (H1 refuted), but depended on maxillary impaction movement (H2) as well as the individual divergence profile (H3). We also demonstrate that changes in labio-mental morphology were not dependent on either the extent of the maxillary advancement or maxillary impaction movement (H4 and H5 refuted). Overall, only divergence caused changes in labio-mental shape. Our findings show that "bone-related" modifications must be distinguished from "labio-mental" modifications and that these neuromuscular adjustments depend on divergence and the surgical maxillary movement induced.

Regarding our morphological analysis of the mandible, our results confirm that LeFort I osteotomy induces post-operative mandibular repositioning (CVA, p <0.0001). This MAP is traditionally defined as being a neuromuscular response to a new occlusion after surgical upper maxillary repositioning [38]. Indeed, this neuromuscular adjustment has been described for over 30 years without any reports defining the cause or how to anticipate it. MAP has to date only been studied in the context of anterior vertical excess associated or not with class II malocclusion. In these studies, maxillary impaction was responsible for mandibular advancement and upward movement [21], but the patients studied were insufficiently characterized. No distinction was made between the type of maxillary movement surgery performed or type of vertical dysmorphosis. In our cohort, homogenous maxillary impaction did not induce significant mandibular rotation. A clockwise maxillary impaction caused mandibular advancement and upward movement, and an anti-clockwise rotation caused only mandibular upward movement. It is therefore necessary to differentiate each maxillary impaction.

The pre- and post-operative mandibular shapes were also correlated with divergence profile. Maxillary surgery induced a symphyseal advancement without associated vertical movement in hypo-divergent patients (CVA pre-op/post-op; p = 0.038) and a symphyseal advancement and upward movement in hyper-divergent patients (CVA pre-op/post-op; p<0.001). There was no significant post-operative change in normo-divergent patients (CVA pre-op/post-op; p = 0.74). It is noteworthy that maxillary surgery in a normo-divergent patient did not induce a clear change in mandibular position, but it did cause a significant labio-mental shape change. There was consequently a neuromuscular adjustment reflex with no detection of mandibular autorotation. The term "mandibular autorotation" does not therefore seem appropriate for describing this neuromuscular adjustment.

Regarding our morphological analysis of labio-mental shape, our results confirm that LeFort I osteotomy induces a change in the soft labio-mental tissues (CVA, p <0.0001). Again, only divergence significantly influenced the change observed in lower lip shape. Similar to our conclusions above, it does not seem appropriate to anticipate these labio-mental effects without analyzing divergence. Different teams have however attempted to correlate the maxillary impaction movements with the movements of cephalometric points [21]; no convincing conclusions were reached. Heterogeneity in data in the literature shows methodological inadequacy and/or the absence of comparability between the patient cohorts studied.

We describe here that advancement of the bony chin point paradoxically causes the soft tissue of the chin to be pushed back. This dissociation between mandibular movements and their cutaneous effects can be explained by the contraction of the labio-mental muscle complex. In fact, mobilization of bone structures induces movement in all the muscular-cutaneous tissues. The muscles change shape, contract or relax, and the tension inevitably impacts skin appearance. We think that advancement of the bony chin point induces a reflex contraction in the mentalis muscle, which is responsible for an upper movement in the soft tissue of the chin and an eversion in lip vermilion. The mentalis muscle acts as: (1) a levator muscle for the skin on the chin, causing wrinkling, and (2) an indirect levator muscle of the lower lip, projecting it forwards [39, 40]. The morphological labial changes observed in hyper- and hypo-divergent patients suggest that contraction of the mentalis muscle takes place during the post-operative period. In addition, it is interesting to consider that the bone insertion in the mentalis muscles corresponds to bony chin point B. The anatomical descriptions of this muscle confirm that it is inserted deep into the gingivo-labial recess and superficially into the labio-mental fold. It therefore appears logic that moving the bone attachment site back induces an inevitable receding of the skin (observed in the normo-divergent group), but advancement of the bone attachment site induces a reflex contraction and thus has little effect on chin position. In this light, it would be worth investigating short and long-term muscular responses to verify whether or not early muscular contraction balances itself out over time after the intervention. Some authors have also suggested that post-operative labio-mental changes are likely secondary to a muscular contraction without conducting more precise analyzes [41, 42].

The limitations of our study owe to the single center and retrospective design that ultimately limit patient inclusion. Inclusion of more patients are required to confirm the effect of clinical explanatory variables studied. In addition, since mandibular autorotation depends on neuromuscular rehabilitation, it would have been interesting to repeat the measurements at different post-operative times to analyse the stability in changes observed. Indeed, some authors have suggested performing a postoperative electromyogram to study muscle contraction secondary to bone movements after mandibular osteotomy [38, 43]. This methodology could be suitable for validating (or disproving) our hypotheses. Finally, this study could have been improved by performing the same multivariate statistical analyzes based on three-dimensional reconstruction, which is limited by the need for postoperative CT scans. Our routine post-operative clinical follow-up does not include CT scans, but we wish to invest in photogrammetry which would enable us to continue our soft tissue analysis at a lower cost and without subjecting the patient to irradiation.

This is the first study denoting post-operative changes in mandibular-labio-mental morphology after maxillary surgery according to different clinical and surgical variables. It is also the first time that geometric morphometric has been used to characterise and explain the neuromuscular adjustment of the chin to mandibular movement. Our data therefore strengthens the idea that mandibular-mentonal neuromuscular readjustment cannot be reduced to a simple mandibular autorotation movement. We think that understanding this mechanism requires a better characterization of the patients included (notably divergence) in the former published studies. For instance in our study, we could consider it inadequate to analyse mandibular repositioning in a cohort of patients not presenting with the same sagittal malocclusion. Given that skeletal malocclusion can be due to cranio-facial growth disorders, we can assume that a patient presenting with class II malocclusion will not have the same muscular development as a patient with class III malocclusion, and therefore potentially experience a different neuromuscular response to surgical repositioning of the maxilla. There are also many other confounding factors that can influence the muscular response which require investigation in future research, such as phenotypic sub-groups, sex, and post-operative follow-up.

## Conclusion

Maxillary advancement alone does not result in mandibular or labiomental changes and so the clinical indication of maxillary surgery alone for the correction of class III dentoskeletal dsymorphoses can be justified. Surgical maxillary rotation results in mandibular upper movement associated with varying degrees of advancement, the extent of which depends on maxillary rotation movement and the divergence of the patient. However, this mandibular movement would not have a significant impact on labio-mentonal projection, morphological criterion of patient concern.

The surgical procedure therefore does not have the same bone-related and musculocutaneous effects on patients with the same class III malocclusion. It is therefore essential for surgeons to understand the effects of their procedures on musculocutaneous tissues to anticipate post-operative outcomes. Overall, we can conclude that the term "mandibular autorotation" is not appropriate for describing these complex neuromuscular adjustments and further studies are needed to understand this phenomenon.

## Supporting information

S1 Text (TXT)

S2 Text (TXT)

S3 Text (TXT)
